# Every Man His Own Dentist

**Published:** 1859-02

**Authors:** 


### EVERY MAN HIS OWN DENTIST.

We have been favored with a copy of a neat pamphlet of 24 pages, bearing the above caption, which, as the title page informs us, is "A Treatise on the proper management of the

Teeth, with full instructions to preserve and keep them beautiful, to the most extreme old age, without the aid of the Dentist; with instructions by which to detect quack dentists, and the propriety of avoiding patronising the same. By H. H. Black, Surgeon Dentist." Price, 25 cents.

We have hurriedly glanced over this production, and find in it much interesting information and many valuable hints and suggestions, which, if properly applied, will go far toward the object aimed at; but we think the Doctor—in giving "instructions to *preserve and keep the teeth beautiful to the most extreme old age*"—claims a little too much—more than can be accomplished in one generation,—while much may be done. There is undoubtedly a large amount of caries induced by constitutional causes, transmitted from parent to child, which will require the coöperative skill of the dentist and physician, with the most vigilant care of parents, for several successive generations; but a beginning in the right direction can not be made too soon, the community can not become too soon nor too well informed on this subject. If it were the loss of the teeth ONLY, it would not be so bad; but comfort, health, and beauty are involved. No person can long enjoy either of those blessings, without good teeth. We hope this little Book will have a large circulation and be the means of doing much good.

